# Copper metabolism of astrocytes

**DOI:** 10.3389/fnagi.2013.00009

**Published:** 2013-03-14

**Authors:** Ralf Dringen, Ivo F. Scheiber, Julian F. B. Mercer

**Affiliations:** ^1^Centre for Biomolecular Interactions Bremen, University of BremenBremen, Germany; ^2^Centre for Environmental Research and Sustainable Technology, University of BremenBremen, Germany; ^3^Department of Parasitology, Faculty of Science, Charles UniversityPrague, Czech Republic; ^4^Centre for Cellular and Molecular Biology, School of Life and Environmental Sciences, Deakin UniversityBurwood, VIC, Australia

**Keywords:** ATP7A, astroglia, copper export, Ctr1, metallothioneins, oxidative stress, toxicity, transport

## Abstract

This short review will summarize the current knowledge on the uptake, storage, and export of copper ions by astrocytes and will address the potential roles of astrocytes in copper homeostasis in the normal and diseased brain. Astrocytes in culture efficiently accumulate copper by processes that include both the copper transporter Ctr1 and Ctr1-independent mechanisms. Exposure of astrocytes to copper induces an increase in cellular glutathione (GSH) content as well as synthesis of metallothioneins, suggesting that excess of copper is stored as complex with GSH and in metallothioneins. Furthermore, exposure of astrocytes to copper accelerates the release of GSH and glycolytically generated lactate. Astrocytes are able to export copper and express the Menkes protein ATP7A. This protein undergoes reversible, copper-dependent trafficking between the *trans*-Golgi network and vesicular structures. The ability of astrocytes to efficiently take up, store and export copper suggests that astrocytes play a key role in the supply of neurons with copper and that astrocytes should be considered as target for therapeutic interventions that aim to correct disturbances in brain copper homeostasis.

## Introduction

Astrocytes have many important functions in the brain, including the maintenance of extracellular ion homeostasis, the modulation of synaptic transmission and plasticity, the supply of metabolites, and the defense of the brain against oxidative stress and toxins (Parpura et al., 2012; Schmidt and Dringen, 2012). In addition, astrocytes are considered as key regulators of the homeostasis of the redox-active metals iron and copper in the brain (Dringen et al., 2007; Tiffany-Castiglioni et al., 2011; Scheiber and Dringen, 2013). Copper is essential for brain cells as a cofactor and structural component of various enzymes that are involved in important biochemical pathways such as the respiratory chain, the antioxidative defense and the iron metabolism (Scheiber and Dringen, 2013). However, excess of copper in cells is harmful, since copper in redox-active form can catalyze the production of hydroxyl radicals in a Fenton-like reaction, thereby inducing oxidative stress and cell damage (Halliwell and Gutteridge, 2007). Thus, a tight regulation of cellular copper metabolism is required in order to ensure sufficient availability of copper for essential enzymes without concomitant copper-induced oxidative damage.

The presence of many copper-containing enzymes in astrocytes (Scheiber and Dringen, 2013) demonstrates that brain astrocytes require copper as essential element. Histochemical staining of brain sections revealed that copper is present in astrocytes in normal brain (Szerdahelyi and Kasa, 1986). Elevated astrocytic copper levels have been found in the brains of North Ronaldsay sheep, an animal model for copper toxicosis (Haywood et al., 2008). Astrocytes have been reported to be remarkably resistant against copper-induced toxicity (Chen et al., 2008; Reddy et al., 2008; Scheiber and Dringen, 2013). Thus, these cells appear to be equipped with the machinery to deal well with even large amounts of copper. This short review will summarize the current knowledge on the uptake, storage and export of copper in cultured astrocytes and will describe alterations of basal metabolic pathways of astrocytes after exposure to copper. Finally, we will address the potential roles that astrocytes may play in the copper homeostasis of the normal brain and in the dysregulation of copper homeostasis that has been connected with a number of human diseases.

## Uptake storage and export of copper

### Copper uptake

**Figure 1:**
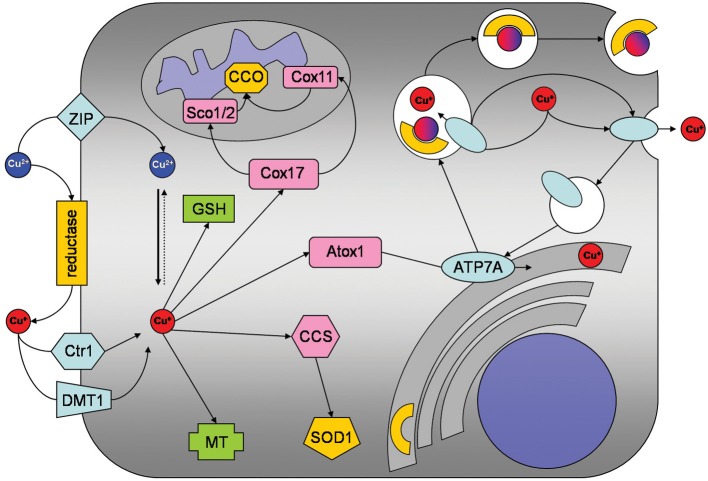
**Copper metabolism of astrocytes.** Copper is taken up into astrocytes by the copper transporter receptor 1 (Ctr1) and also by Ctr1-independent mechanisms which may include the divalent metal transporter 1 (DMT1) or members of the ZIP family of metal transporters. An ecto-cuprireductase and/or extracellular ascorbate will provide the reduced copper species for astrocytic uptake by Ctr1 or DMT1. Accumulated copper is stored in astrocytes as complex with GSH or in metallothioneins (MT). In addition, copper is shuttled to its specific cellular target, by the copper chaperones CCS to superoxide dismutase 1 (SOD1), by Cox17 to Sco1/2 and Cox11 for subsequent incorporation into cytochrome *c* oxidase and by antioxidant protein 1 (Atox1) to ATP7A. ATP7A transports copper into the *trans*-Golgi network for subsequent incorporation into copper-dependent enzymes. When the cellular copper level rises above a certain threshold, ATP7A translocates reversibly via vesicles to the plasma membrane to export copper.

Several groups have reported that cultured astrocytes efficiently accumulate copper (Brown, 2004; Scheiber et al., 2010a,b, 2012; Qian et al., 2012). The apparent K_M_ value for copper uptake of about 10 μM as well as the expression of the copper transporter receptor 1 (Ctr1) in cultured astrocytes (Scheiber et al., 2010a) suggest that this transporter is responsible for the high affinity copper uptake into astrocytes (Figure 1). However, the inhibition of copper accumulation in astrocytes by zinc (Scheiber et al., 2010a,b) suggests that Ctr1-independent transport processes also contribute to the astrocytic copper accumulation. Transporters that could mediate Ctr1-independent copper uptake into astrocytes include the divalent metal transporter 1 (DMT1) and members of the Zrt/IRT-like protein (ZIP) family (Scheiber et al., 2010a; Scheiber and Dringen, 2013). Since Cu^+^ has been reported to be the copper species transported by both Ctr1 and DMT1 (Lee et al., 2002; Arredondo et al., 2003), an ecto-cuprireductase on the plasma membrane of astrocytes and/or astrocyte-derived ascorbate (Scheiber and Dringen, 2013) may be involved in reducing extracellular Cu^2+^ to the Cu^+^ that is subsequently taken up into astrocytes (Figure 1). An additional protein that has been suggested to contribute to the astrocytic copper uptake or export is the prion protein (Brown, 2004).

### Storage and intracellular trafficking of copper

**Figure 2:**
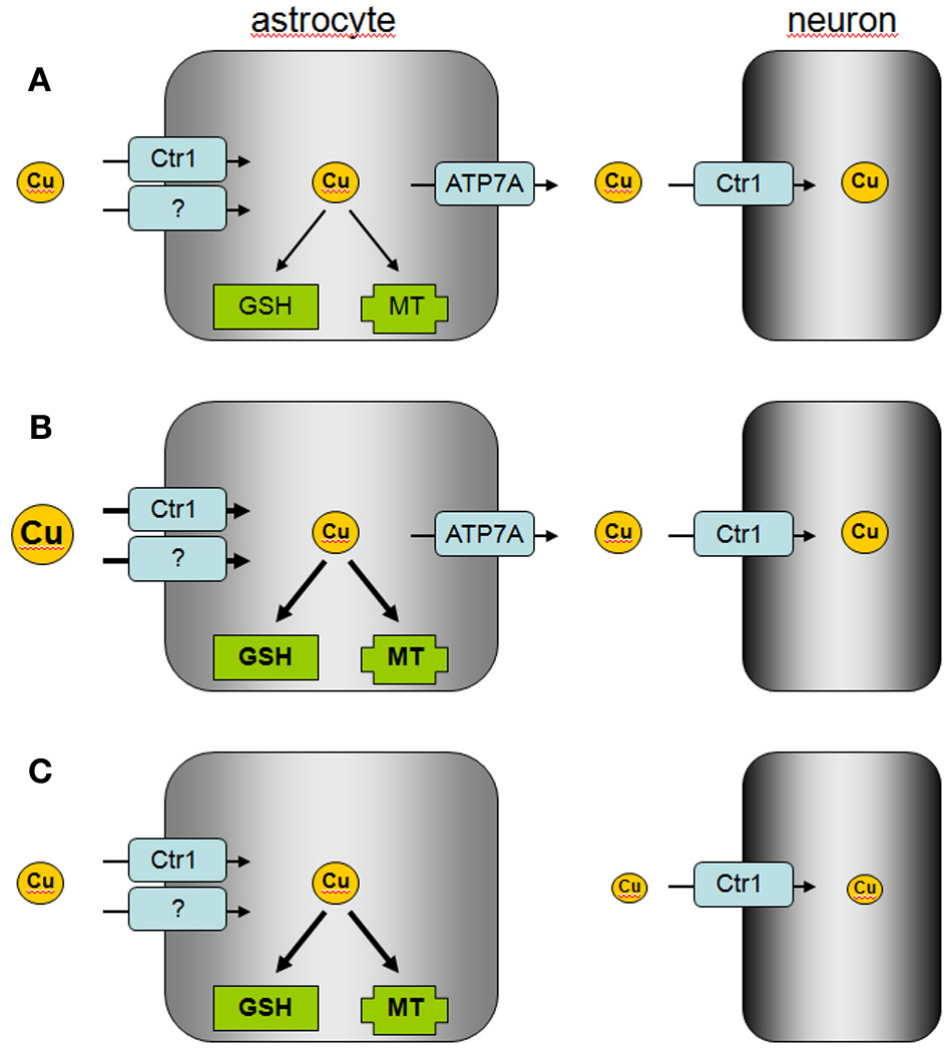
**Proposed model of astrocytic copper supply to neurons.(A)** In the normal brain, copper is efficiently taken up by astrocytes via Ctr1 and other transporters which are so far not identified. Excess of copper is stored by astrocytes in MT or as GSH complex and copper is released via ATP7A to supply neurons with copper. **(B)** In copper overload conditions, excess of copper is efficiently taken up into astrocytes and stored in MT or as GSH complex to prevent copper-induced neurotoxicity. **(C)** In Menkes disease, astrocytes accumulate and store copper, but the mutated ATP7A is unable to mediate astrocytic copper export and copper supply to neurons.

Despite of their efficient accumulation of exogenous copper, astrocytes have been reported to be remarkably resistant to copper-induced toxicity (Chen et al., 2008; Reddy et al., 2008; Scheiber and Dringen, 2013). The most likely reason for this resistance is that astrocytes have a high capacity to store excess copper as complex with glutathione (GSH) and in metallothioneins (MT; Figures 1, 2) which keep the excess copper bound in a non-toxic form. When exposed to copper cultured astrocytes elevate their cellular levels of GSH (Scheiber and Dringen, 2011a) and MT (Scheiber and Dringen, 2013), thereby increasing their storage capacity for copper.

For incorporation into copper-dependent enzymes, copper is shuttled to its target proteins by specific copper chaperones (Robinson and Winge, 2010). The copper chaperone for Cu/Zn superoxide dismutase (CCS) that delivers copper to Cu/Zn superoxide dismutase is expressed in astrocytes (Rothstein et al., 1999). In contrast, expression of Atox1 and Cox17, which deliver copper to ATP7A and to cytochrome c oxidase, respectively, has not been reported so far for astrocytes. However, as astrocytes express functional ATP7A (Scheiber et al., 2012) and cytochrome c oxidase (Bolaños and Heales, 2010), Atox1 and Cox17 are likely to be expressed in these cells.

### Export of copper

Cellular export of copper is mediated by the copper ATPases ATP7A and ATP7B which are mutated in the human disorders Menkes disease and Wilson's disease, respectively (Huster, 2010; Kaler, 2011). Cultured astrocytes have recently been shown to export copper in a time-, concentration- and temperature-dependent manner (Scheiber et al., 2012). This copper export is most likely mediated by ATP7A (Figure 1) as this protein is expressed in astrocytes in culture and in brain (Barnes et al., 2005; Niciu et al., 2007; Scheiber et al., 2012). The copper-dependent trafficking of ATP7A between the *trans*-Golgi network and sites close to the plasma membrane (Scheiber et al., 2012) strongly suggests a contribution of ATP7A in copper export from astrocytes. This view is also supported by the marked accumulation of copper in astrocytes derived from the macular mouse model of Menkes disease (Kodama et al., 1991). The potential of astrocytes to export copper suggests that these cells can supply other types of brain cells with copper and that impaired copper supply from astrocytes contributes to the neuronal copper deficiency in Menkes disease (Figure 2).

## Modulation of astrocytic metabolism by copper

Elevation of the cellular copper content of cultured astrocytes caused a time- and concentration-dependent acceleration in glucose consumption and lactate release (Scheiber and Dringen, 2011b). This consequence of copper treatment appears not to be mediated by mitochondrial impairment, but was prevented by inhibition of protein synthesis (Scheiber and Dringen, 2011b). Thus, the copper-induced stimulation of glycolytic flux in astrocytes is likely to depend on transcription and/or translation. Copper-activated transcription factors such as the nuclear factor kappa B or the metal transcription factor 1 have been suggested to contribute to the copper-induced stimulation of glycolytic flux in astrocytes (Scheiber and Dringen, 2013).

Elevated copper content also affects the GSH metabolism of astrocytes. Exposure to copper chloride markedly increased the cellular GSH content of cultured astrocytes (Scheiber and Dringen, 2011a). Copper-induced increase in γ-glutamate cysteine ligase activity by a post-translational mechanism and increased uptake of the GSH precursors cysteine or cysteine into astrocytes have been suggested as potential reasons for the increased specific GSH content of copper-treated astrocytes (Scheiber and Dringen, 2011a, 2013). A compromised GSH export which has been reported to increase cellular GSH contents in astrocytes (Minich et al., 2006) can be excluded as a contributor to the increased cellular GSH content of copper-treated astrocytes, as the GSH export is accelerated in copper-loaded astrocytes (Scheiber and Dringen, 2011a). This accelerated, temperature-sensitive GSH export is mediated by the multidrug resistance protein 1 and is considered to be a direct consequence of the increased cellular GSH concentration in copper-treated astrocytes (Scheiber and Dringen, 2011a).

## Functions of astrocytes in the copper metabolism of normal and diseased brain

The ability of astrocytes to efficiently take up, store and export copper suggests that these cells serve as key regulators of the copper homeostasis in the brain (Tiffany-Castiglioni et al., 2011; Scheiber and Dringen, 2013). Efficient copper uptake and storage in astrocytes serves to protect other brain cells against copper toxicity. The known high antioxidative potential of astrocytes (Dringen et al., 2005) is likely to protect these cells from acute copper toxicity, while the upregulation of the cellular copper storage capacity by induction of MT synthesis (Scheiber and Dringen, 2013) and by elevated GSH synthesis (Scheiber and Dringen, 2011a) will contribute to the prolonged resistance of astrocytes against copper-induced toxicity. In addition, the ability of astrocytes to export copper suggests that astrocytes are able to provide copper to neurons and other neighboring cells in the brain (Figure 2). Further studies are required to investigate how signals derived from neurons and/or other types of brain cells will affect the basal copper metabolism of astrocytes and whether such signals could modulate the copper export from astrocytes.

Disturbances in copper homeostasis in brain occur in both Menkes disease and Wilson's disease (Kaler, 2011; Kodama et al., 2011), but also in other neurodegenerative disorders such as Alzheimer's disease and Parkinson's disease (Rivera-Mancia et al., 2010; Double, 2012; Greenough et al., 2013; Scheiber and Dringen, 2013). The role of astrocytes in the copper homeostasis of the brain in these disorders should be considered. The impaired supply of copper from astrocytes to neurons due to mutations of the Menkes protein ATP7A may foster the neuronal copper deficiency in Menkes disease, while the capacity of astrocytes to uptake and store excess of copper may be insufficient to protect neurons against the excess of copper present in the brain in Wilson's disease (Figure 2). In addition, the disturbances in the distribution of brain copper in Alzheimer's disease (Greenough et al., 2013) may reflect impaired supply of copper from astrocytes to neurons (Scheiber and Dringen, 2013). The ability of astrocytes to efficiently accumulate, store and export copper makes this brain cell type to an interesting target for therapeutic strategies that aim to correct the observed disturbances in copper metabolism in human disorders (Greenough et al., 2013; Liddell et al., 2013).

### Conflict of interest statement

The authors declare that the research was conducted in the absence of any commercial or financial relationships that could be construed as a potential conflict of interest.
